# Supplementary material for: Identification and functional analysis of non-coding regulatory small RNA FenSr3 in Bacillus amyloliquefaciens LPB-18
Source: PeerJ. 2023 May 15;11:e15236. doi: 10.7717/peerj.15236 (PMC10194069; doi:10.7717/peerj.15236)
Supplement: Supplemental Information 4 [file peerj-11-15236-s004.zip › KO/CK-vs-T1_map/map05211.html]

KEGG PATHWAY: Renal cell carcinoma - Reference pathway


|  |  |
| --- | --- |
| **Renal cell carcinoma - Reference pathway** |  |

[
Pathway menu
| Organism menu
| Pathway entry
| Show description
| User data mapping
]

Renal cell cancer (RCC) accounts for ~3% of human malignancies and its incidence appears to be rising. Although most cases of RCC seem to occur sporadically, an inherited predisposition to renal cancer accounts for 1-4% of cases. RCC is not a single disease, it has several morphological subtypes. Conventional RCC (clear cell RCC) accounts for ~80% of cases, followed by papillary RCC (10-15%), chromophobe RCC (5%), and collecting duct RCC (<1%). Genes potentially involved in sporadic neoplasms of each particular type are VHL, MET, BHD, and FH respectively. In the absence of VHL, hypoxia-inducible factor alpha (HIF-alpha) accumulates, leading to production of several growth factors, including vascular endothelial growth factor and platelet-derived growth factor. Activated MET mediates a number of biological effects including motility, invasion of extracellular matrix, cellular transformation, prevention of apoptosis and metastasis formation. Loss of functional FH leads to accumulation of fumarate in the cell, triggering inhibition of HPH and preventing targeted pVHL-mediated degradation of HIF-alpha. BHD mutations cause the Birt-Hogg-Dube syndrome and its associated chromophobe, hybrid oncocytic, and conventional (clear cell) RCC.

|  |  |  |
| --- | --- | --- |
| Reference pathway | 184% 150% 122% 100% 82% 67% 55% | 图片下载 |
